# Chasing sleep physicians: ChatGPT-4o on the interpretation of polysomnographic results

**DOI:** 10.1007/s00405-024-08985-3

**Published:** 2024-10-20

**Authors:** Christopher Seifen, Tilman Huppertz, Haralampos Gouveris, Katharina Bahr-Hamm, Johannes Pordzik, Jonas Eckrich, Harry Smith, Tom Kelsey, Andrew Blaikie, Christoph Matthias, Sebastian Kuhn, Christoph Raphael Buhr

**Affiliations:** 1 Sleep Medicine Center & Department of Otolaryngology, Head and Neck Surgery, University Medical Center Mainz, Mainz, Germany; 2 School of Computer Science, University of St Andrews, St Andrews, UK; 3 School of Medicine, University of St Andrews, St Andrews, UK; 4 Institute for Digital Medicine, University Hospital of Giessen and Marburg, Philipps-University Marburg, Marburg, Germany

**Keywords:** ChatGPT, ChatGPT-4o, Large language models, Artificial intelligence, Obstructive sleep apnea, OSA, Polysomnography, PSG, Digital health, Sleep medicine

## Abstract

**Background:**

From a healthcare professional's perspective, the use of ChatGPT (Open AI), a large language model (LLM), offers huge potential as a practical and economic digital assistant. However, ChatGPT has not yet been evaluated for the interpretation of polysomnographic results in patients with suspected obstructive sleep apnea (OSA).

**Aims/objectives:**

To evaluate the agreement of polysomnographic result interpretation between ChatGPT-4o and a board-certified sleep physician and to shed light into the role of ChatGPT-4o in the field of medical decision-making in sleep medicine.

**Material and methods:**

For this proof-of-concept study, 40 comprehensive patient profiles were designed, which represent a broad and typical spectrum of cases, ensuring a balanced distribution of demographics and clinical characteristics. After various prompts were tested, one prompt was used for initial diagnosis of OSA and a further for patients with positive airway pressure (PAP) therapy intolerance. Each polysomnographic result was independently evaluated by ChatGPT-4o and a board-certified sleep physician. Diagnosis and therapy suggestions were analyzed for agreement.

**Results:**

ChatGPT-4o and the sleep physician showed 97% (29/30) concordance in the diagnosis of the simple cases. For the same cases the two assessment instances unveiled 100% (30/30) concordance regarding therapy suggestions. For cases with intolerance of treatment with positive airway pressure (PAP) ChatGPT-4o and the sleep physician revealed 70% (7/10) concordance in the diagnosis and 44% (22/50) concordance for therapy suggestions.

**Conclusion and significance:**

Precise prompting improves the output of ChatGPT-4o and provides sleep physician-like polysomnographic result interpretation. Although ChatGPT shows some shortcomings in offering treatment advice, our results provide evidence for AI assisted automation and economization of polysomnographic interpretation by LLMs. Further research should explore data protection issues and demonstrate reproducibility with real patient data on a larger scale.

**Supplementary Information:**

The online version contains supplementary material available at 10.1007/s00405-024-08985-3.

## Introduction

With the introduction of artificial intelligence (AI) large language models (LLMs) healthcare professionals are experiencing a technical revolution. Launched in November 2022 by OpenAI Inc., Chat Generative Pre-Trained Transformer (ChatGPT) has become one of the most popular LLMs [1]. ChatGPT has garnered interest for its capability to analyze and convert textual and graphical inputs into text-based outputs, simulating human-like conversations and generating human-like content [2]. The inclusion of AI, such as ChatGPT, in routine clinical practice holds great potential: from the identification of research topics to support in routine diagnostic processes, such as the analysis and interpretation of medical data sets, to the development of personalized therapy suggestions. In addition, AI can improve the situation of patients by providing them with easily accessible and understandable information. However, current shortcomings, limitations, and barriers to introducing LLMs in clinical practice include concerns about data privacy, accuracy and reliability of AI-generated insights, integration with existing medical systems, and the need for rigorous validation and regulation to ensure patient safety [3, 4]. ChatGPT has attracted more than a billion users in a remarkably short time, and a PubMed search on May 30, 2024 returned more than 3300 ChatGPT-related results, demonstrating the huge interest in this technology in the medical field.

The implementation of ChatGPT in the field of sleep medicine has recently been successfully tested, e.g. in the processing of patient questions on obstructive sleep apnea (OSA) from everyday clinical practice and in the evaluation of questions on OSA-specific surgeries [5–7]. OSA is the most common type of sleep-disordered breathing with increasing prevalence in the general adult population around the globe [8, 9]. OSA is characterized by upper airway collapses that clinically result in daytime sleepiness and fatigue [10]. The association with serious comorbidities such as arterial hypertension [11], coronary artery disease [12], or stroke [13] make OSA a major public health concern. Its diagnostic approach includes home sleep apnea testing (HSAT) or full-night polysomnography (PSG) in a sleep laboratory. The first-line therapy of moderate and severe OSA is the use of positive airway pressure (PAP) [14, 15].

Current clinical practice requires that HSAT or polysomnographic data are evaluated by experienced sleep medicine specialists. This procedure ensures high quality and therefore safety for the patient, but is time-consuming and requires trained personnel. In this context, ChatGPT could represent a valuable tool, e.g. for evaluating sleep medicine data or interpreting it in selected cases. However, it is currently uncertain whether ChatGPT can interpret polysomnographic results in such a way that a correct diagnosis is made and a guideline-oriented therapy suggestion is given. As we are convinced that LLMs, e.g. in the form of ChatGPT, will sooner or later be implemented in everyday clinical practice, this proof-of-concept study was designed to test whether ChatGPT is fundamentally capable of accurately evaluating selected data from fictitious polysomnographic results.

## Material and methods

For this study, we generated *n* = 40 fictitious polysomnographic results from* n* = 40 consecutive fictitious patients. Patients 1–30 were designed to be simple cases, while patients 31–40 were more complex.

We defined the following polysomnographic parameters for each of the 40 fictitious patients:

- age,
- sex,
- body mass index (BMI),
- apnea hypopnea index (AHI, apneas and hypopneas per hour of sleep),
- apnea index (AI, apneic events per hour of sleep),
- hypopnea index (HI, hypopnea events per hour of sleep),
- cumulative apnea and hypopnea duration during sleep,
- oxygen desaturation index (ODI, desaturation episodes as a decrease in mean oxygen saturation of ≥ 3% per hour of sleep),
- average oxygen saturation,
- percentage of cumulative time with oxygen saturation below 90% during sleep (T90),
- total sleep time (TST),
- sleep efficacy (ratio total sleep time to time in bed), and
- ratio AHI in supine position to not supine position.

**Fig. 1:**
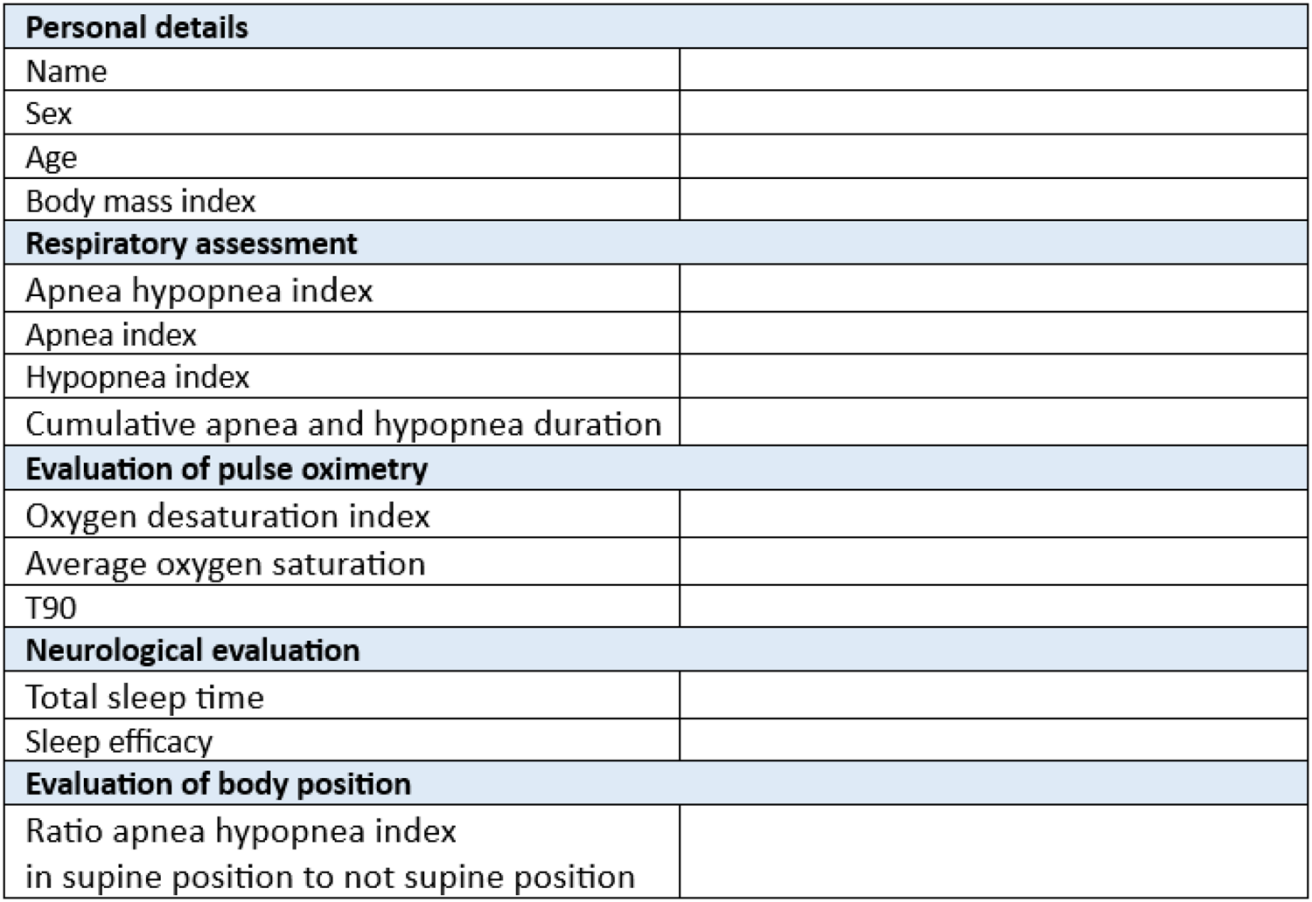
The template used to generate fictitious polysomnographic results. T90: percentage of cumulative time with oxygen saturation below 90% during sleep

Polysomnography is a standardized examination and its results are presented in a standardized format. For this study, we followed the format of the institutions’ own accredited sleep laboratory. Figure 1 shows the template we used to generate the fictitious polysomnographic results. The detailed polysomnographic results of all 40 fictitious patients can be found in the supplemental materials.

The following general assumptions applied to patients 1–30 (simple cases) in order to minimize important confounders:

- first time polysomnographic recording due to assumed OSA-typical clinical complaints,
- the polysomnographic recording was performed technically correct in an accredited sleep laboratory under the supervision of a licensed technician,
- the polysomnographic recording had no technical problems and was not interrupted,
- no presence of comorbidities of any kind,
- no intake of medication of any kind,
- no sleep-disordered breathing other than OSA (e.g. periodic breathing or Cheyne-Stokes breathing), and
- the patient slept in all positions, with at least 60 min of the total sleep time spent on the back.

**Fig. 2:**
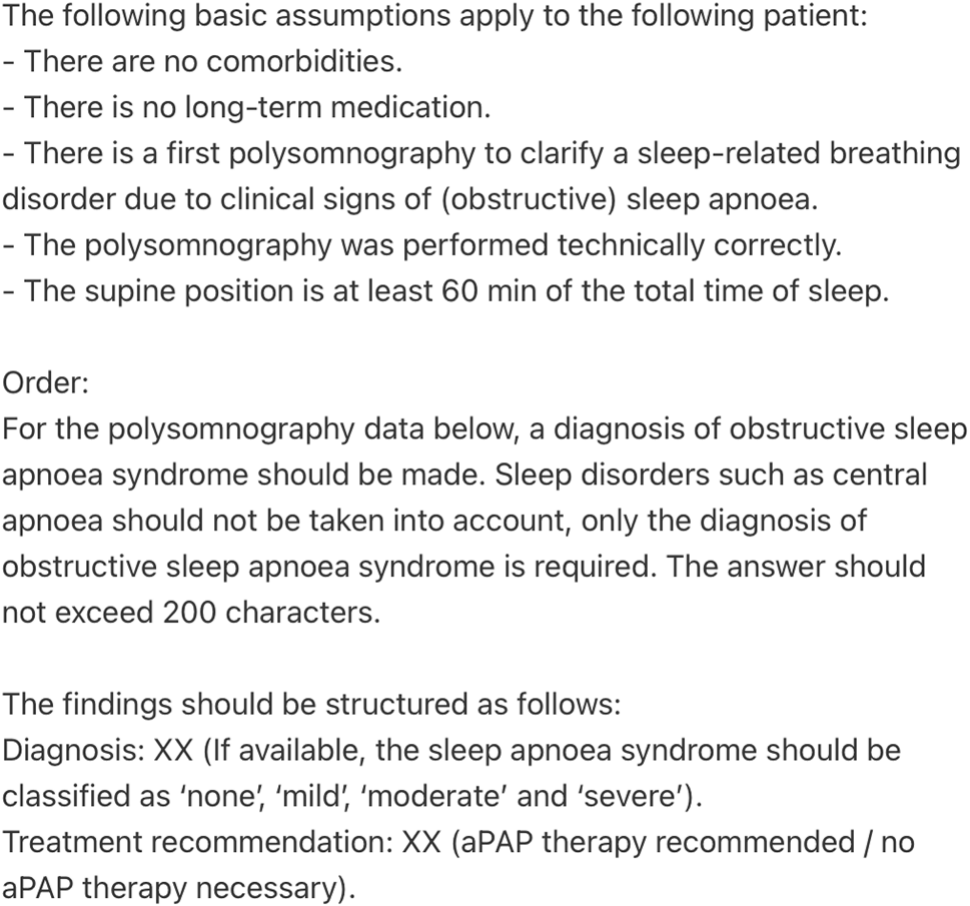
Prompt for patients 1–30, translated by DeepL (Cologne, Germany)

After testing different prompts, the polysomnographic data of patients 1–30 were passed to ChatGPT-4o (latest version) using the prompt shown in Fig. 2. ChatGPT-4o was given the general assumptions as mentioned before, asked to make a diagnosis and decide whether automatic PAP (aPAP) therapy is necessary for each fictitious patient based on their polysomnographic data. In each case, ChatGPT-4o was asked to use a maximum of 200 characters for the answer. Consequently, for patients 1–30 the prompt of Fig. 2 followed by the patients specific table (as text data) exemplary illustrated in Fig. 1 was passed to ChatGPT-4o.

For patients 31–40 (more complex cases) the following general assumptions were assumed in order to minimize important confounders:

- no presence of comorbidities of any kind,
- no intake of medication of any kind,
- at least mild obstructive sleep apnoea already confirmed by external polysomnography/polygraphy,
- the external polysomnography/polygraphy was performed due to clinical signs of (obstructive) sleep apnea,
- PAP therapy has already been initiated and trialed,
- PAP therapy was not tolerated and therefore rejected by patient,
- various mask fits have already been trialed (e.g. full-face mask, nasal mask, nasal cushion mask),
- presentation in our sleep laboratory for renewed polysomnography,
- the polysomnography was performed technically correctly, and
- the patient slept in all positions, with at least 60 min of the total sleep time spent on the back.

**Fig. 3:**
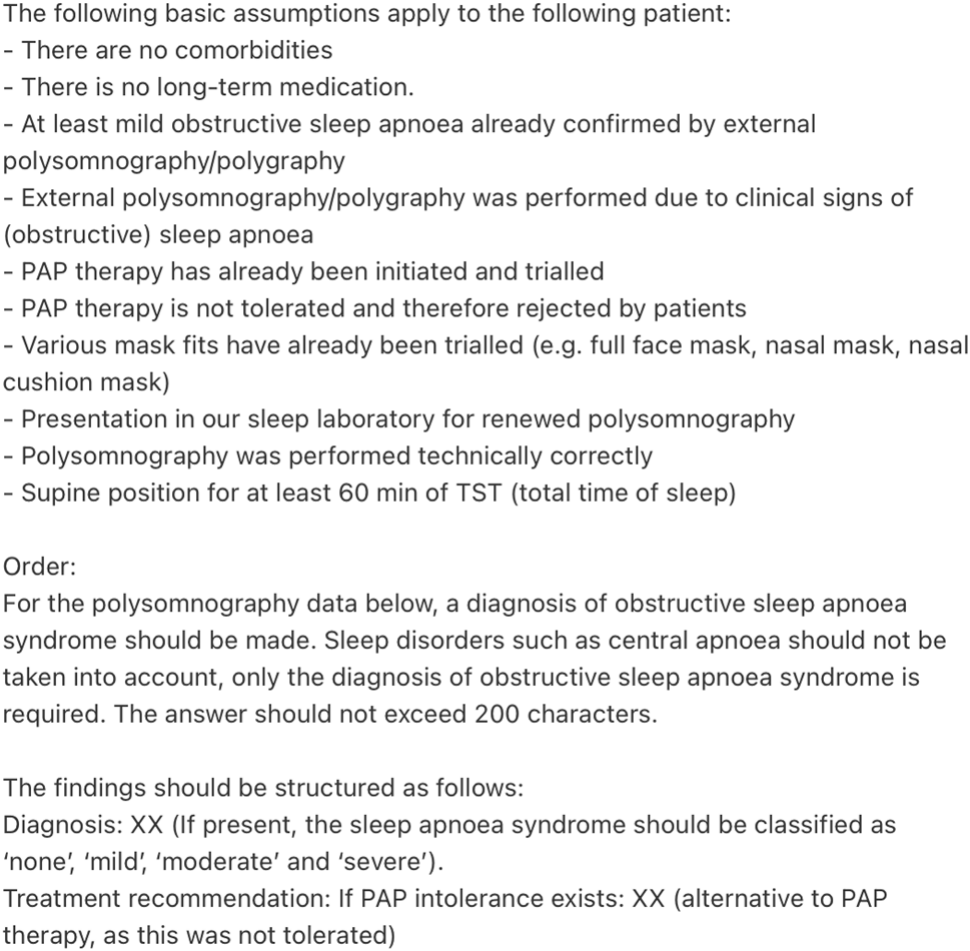
Prompt for patients 31–40, translated by DeepL (Cologne, Germany)

Thus, the prompt was slightly adjusted for patients 31–40 with PAP intolerance, see Fig. 3. ChatGPT-4o was given the general assumptions as mentioned before, asked to make a diagnosis and provide a therapy alternative for each fictitious patient based on their polysomnographic data. In each case, ChatGPT-4o was asked to use a maximum of 200 characters for the answer. Again, for patients 31–40 the prompt of Fig. 3 followed by the patients specific table (as text data) exemplary illustrated in Fig. 1 was passed to ChatGPT-4o.

In a second step, each fictitious polysomnographic result was interpreted by a board-certified sleep physician according to the same general assumptions. Based on the standard guidelines of the American Academy of Sleep Medicine (AASM) [16], a diagnosis was made and an appropriate therapy was suggested. The diagnostic and therapeutic evaluation of the data by the board-certified sleep physician was not submitted to ChatGPT-4o in any of the 40 cases. In all cases, ChatGPT-4o only received the data as shown in Fig. 1.

**Fig. 4:**
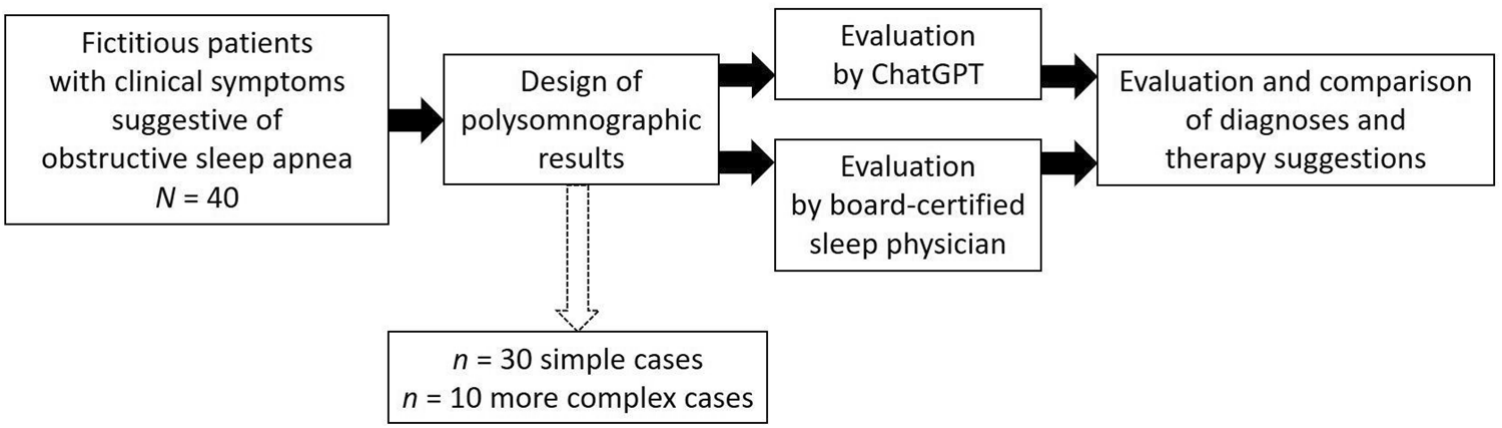
Workflow of the study

In a final step, we compared the diagnosis and therapy suggestions of ChatGPT-4o to those of the sleep physician. Figure 4 shows the workflow of this study.

All data was collected in Microsoft Word and Excel sheets (Microsoft, Redmond, WA, USA). GraphPad Prism version 5.01 (GraphPad Software, Boston, MA, USA) was used for statistical analysis and graphical illustration.

All personal and polysomnographic patient data used in this study are fictitious. They do not correspond to real patient data.

## Results

The complete patient cohort contained 40 adult individuals, 20 (50%) male and 20 (50%) female. The mean age was 50.18 ± 15.31 years, BMI was 28.45 ± 3.64 kg/m2 and AHI was 26.46 ± 19.52/h. According to AASM standard guidelines, three patients had no OSA (AHI < 5/h), twelve patients had mild OSA (AHI ≥ 5/h but < 15/h), ten patients had moderate OSA (AHI 15–30/h) and 15 patients had severe OSA (AHI > 30/h).

In the patient group with no OSA (*n* = 3), the sleep physician did not recommend any specific therapy.

In the patient group with mild OSA (*n* = 12), the sleep physician did not recommend any specific therapy in the simple cases. In the more complex cases, however, therapy suggestions included positional therapy, mandibular protrusion, or OSA surgery (case-specific therapy suggestions can be found in the supplemental material). In the patient group with mild OSA, the highest AHI was 13.8/h.

In the patient group with moderate OSA (*n* = 10), the lowest AHI was 17.5/h and the highest AHI was 29.5/h. For all patients, the sleep physician recommended treatment with aPAP in the pressure range of 5–9 millibar (mbar), except for one patient with a BMI of 32 kg/m2, who was recommended to use the pressure range of 5–10 mbar. Furthermore, all patients with moderate OSA were indicated for a polysomnographic control of the aPAP settings three months after the start of aPAP therapy.

In the patient group with severe OSA (*n* = 15), the lowest AHI was 30.8/h and the highest AHI was 82.5/h. In all patients with first time polysomnography (simple cases), the sleep physician recommended treatment with aPAP in the pressure range of 5–9 mbar, except for three patients with a BMI of ≥ 32 kg/m2, for whom the pressure range of 5–10 mbar was recommended. Again, polysomnographic control of the aPAP settings were indicated three months after the start of aPAP therapy. In the more complex cases, where former PAP therapy was not tolerated, the sleep physician indicated the evaluation of an alternative therapy by means of neurostimulation of the hypoglossal nerve.

**Fig. 5:**
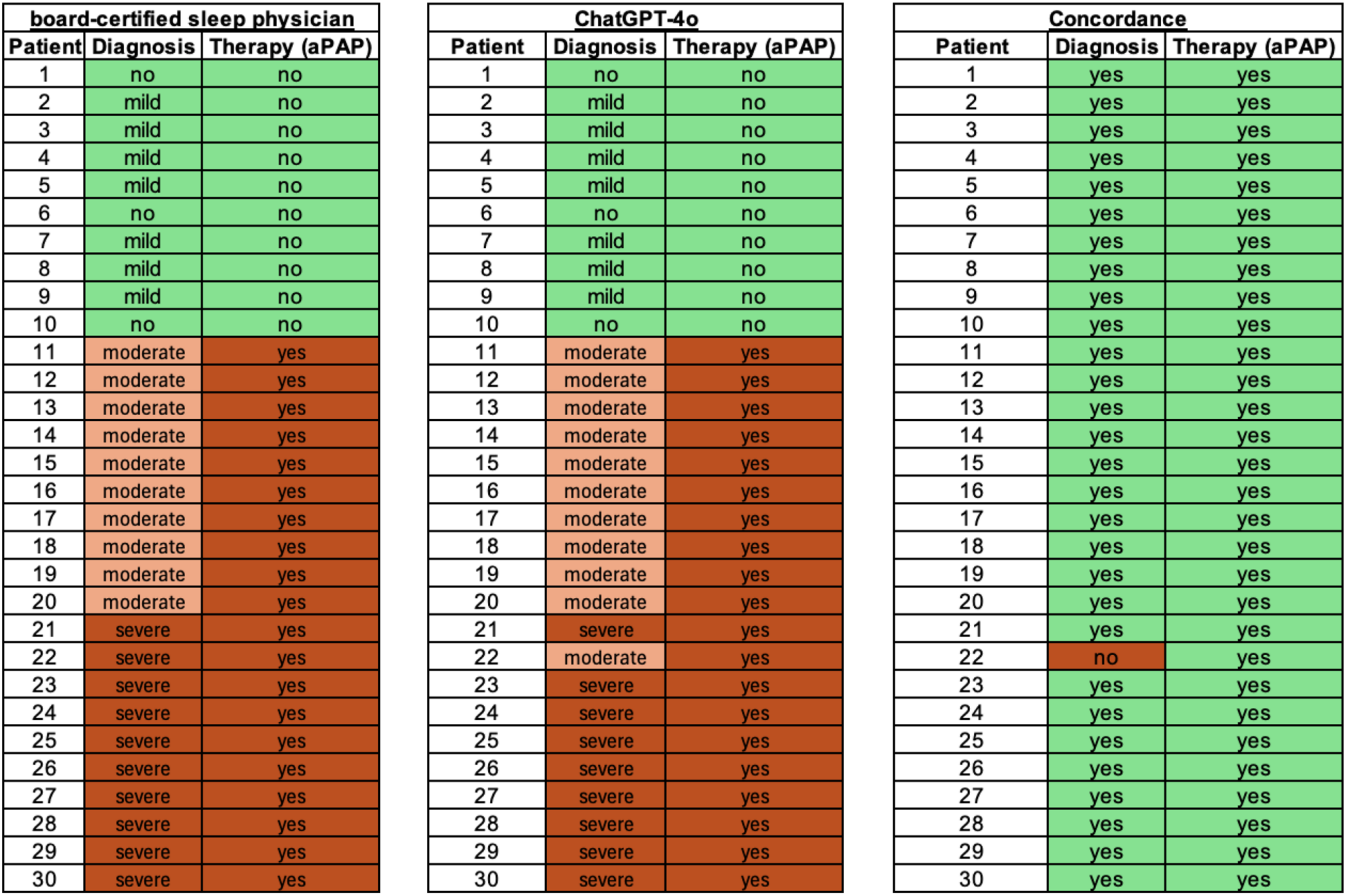
Visualization of diagnosis and therapy suggestions for patients 1–30 as stated by the sleep physician and ChatGPT-4o. Additionally, concordance between the two assessment instances is shown. aPAP: automatic positive airway pressure

As an overview, Fig. 5 shows patients 1–30 (simple cases) with regard to diagnosis and treatment decisions by the sleep physician as well as ChatGPT-4o In addition, the concordance between the two assessment instances is visualized. While the two assessment instances show 100% (30/30) concordance regarding the therapy suggestion, there is a disagreement on a single patient's diagnosis (Patient 22), leading to an overall concordance of 97% (29/30). In this specific case of disagreement, the patient reveals an AHI of 32.0/h, which was incorrectly diagnosed by ChatGPT-4o as a moderate OSA while being rated as severe OSA by the sleep physician according to AASM standard guidelines.

**Fig. 6:**
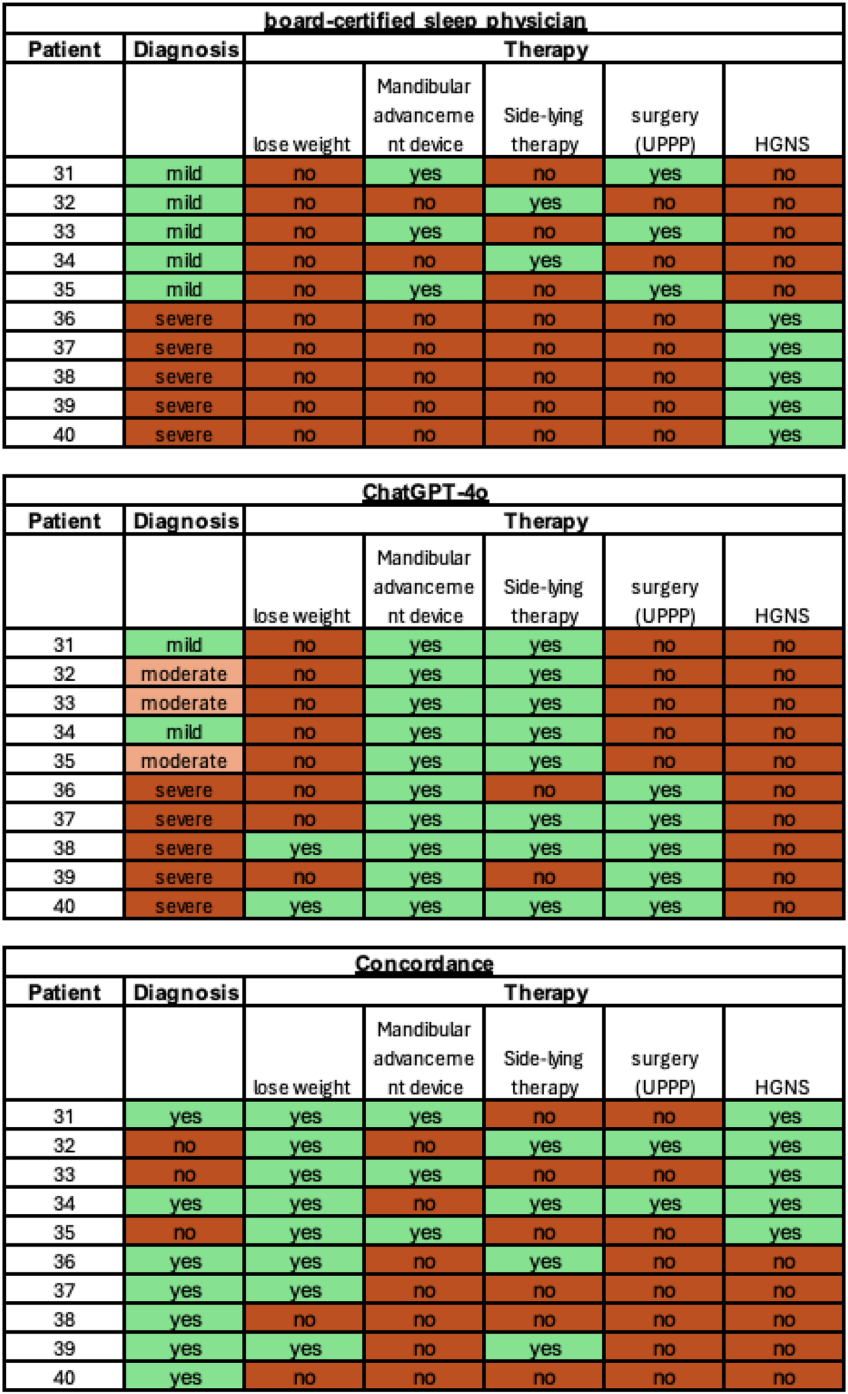
Visualization of diagnosis and therapy suggestions for patients 31–40 (patients with intolerance of therapy with positive airway pressure; more complex cases) as stated by the sleep physician and ChatGPT-4o. Additionally, concordance between the two assessment instances is shown

The patient cases 31–40 (more complex cases) represent patients not tolerating PAP therapy. Diagnosis and therapy suggestions as provided by the sleep physician and ChatGPT-4o as well as the concordance between the two assessment instances are shown in Fig. 6. Regarding the diagnosis, the sleep physician and ChatGPT-4o show 70% (7/10) concordance in the more complex patients with intolerance of PAP therapy. Whereas the sleep physician diagnosed mild OSA for patients 31–35 and severe OSA for patients 36–40, ChatGPT-4o incorrectly diagnosed three patients with a subsequent AHI of 12.4/h, 13.8/h and 12.8/h as moderate OSA. In terms of therapy suggestions, the sleep physician and ChatGPT-4o revealed 44% (22/50) concordance. While the sleep physician recommended no weight loss at all, ChatGPT-4o gave the recommendation for two patients. In reverse, ChatGPT-4o suggested a mandibular advancement device for every patient not tolerating PAP therapy, while the sleep physician recommended this form of therapy for three patients with mild OSA. ChatGPT-4o did not recommend the evaluation of an alternative therapy by means of neurostimulation of the hypoglossal nerve for any patient, while this procedure was suggested for every patient with severe OSA and PAP intolerance by the sleep physician.

## Discussion

Different studies have shown and evaluated useful applications for LLMs in clinical practice [17–32]. However, studies addressing the use of ChatGPT in the interpretation of polysomnographic results are so far lacking. The present proof-of-concept study therefore aimed to investigate the extent to which ChatGPT is able to accurately evaluate selected data from fictitious polysomnographic results of patients with suspected OSA. We provide evidence that ChatGPT may serve as a valuable tool in everyday clinical sleep medicine practice. Regarding the interpretation of fictitious polysomnographic data, ChatGPT provides highly accurate diagnostic and in most cases therapy suggestions that correspond to those of a board-certified sleep physician.

In the field of sleep medicine, ChatGPT was first challenged in November 2023: A prospective cross-sectional study showed that ChatGPT gave the same answers to ten questions on OSA-specific surgical interventions as 97 otolaryngologists with an expertise in OSA in 75% of cases [7]. Another study from December 2023 showed that ChatGPT was able to provide correct answers to questions about OSA in most cases, regardless of the prompting. The answers generated by the LLM were rated as being appropriate for patients [5]. In a third study from February 2024, the five most common questions asked by patients about PAP therapy were answered by sleep medicine specialists and by ChatGPT. The answers were then rated by up to 42 patients and, interestingly, it was found that the specialist's answer was preferred in four out of five cases [6]. The authors of this study explained the result of the study primarily by the fact that ChatGPT can only imitate, but not understand complex social interactions that are based on empathy and direct human contact, including elements such as body language.

Following the key objective of our study, evaluating the quality of ChatGPT-4o´s diagnosis and therapy recommendation of fictitious polysomnographic results, we tested different prompts initially. Prompts based on an open question did provide various different therapy options without a specific recommendation. Thus, we chose a prompt asking for a clear classification of OSA severity and a specific statement whether PAP (e.g. aPAP) therapy as the gold standard first line therapy of moderate to severe OSA is necessary. This setup was meant to provide a diagnosis and treatment recommendation that is as close as possible to that of a board-certified sleep physician. ChatGPT-4o and the board-certified sleep physician revealed 100% (30/30) concordance on recommendations for PAP therapy. Although there were some discrepancies between the two assessment instances in the classification of OSA severity, with only 97% (29/30) agreement, this did not affect the correct therapy suggestions for patients suitable for PAP therapy. Thus, the brief diagnosis and therapy suggestions of ChatGPT-4o for patients 1–30 could have been used literally in clinical practice without a single patient being treated incorrectly.

For patients 31–40 with PAP therapy intolerance, the prompt was modified to an open question asking about alternative treatment strategies, as the gold standard of first line PAP therapy was not applicable. In this context, the board-certified sleep physician and ChatGPT-4o showed a lower agreement of 70% (7/10) for diagnosis and 44% (22/50) for therapy suggestions. Interestingly, ChatGPT-4o did not mention the evaluation of an alternative therapy by neurostimulation of the hypoglossal nerve (hypoglossal nerve stimulation, HGNS) [33]. The neglect of HGNS therapy might be explained by the fact that there is much less literature available regarding this relatively new form of OSA therapy. ChatGPT-4o may lack familiarity with the topic and thus underestimates the relevance of this therapy, due to little training data about HGNS therapy. Selecting a suitable treatment alternative for patients with PAP therapy intolerance is a highly complex procedure. In routine clinical practice, this involves not only the assessment of specific polysomnographic data, but also personal preferences, individual anatomical factors, medical history, existing collaborations with medical supply distributors or available surgical approaches. One of the most important factors may be the clinical experience of the sleep physician.

We acknowledge several limitations of the present study. First, due to data protection aspects, all data used in this study are fictitious and were not collected from real polysomnographic recordings. Although our patient profiles represent a broad and typical spectrum of cases, ensuring a balanced distribution of demographics and clinical characteristics, this circumstance may have influenced the presented results independently. Secondly, general assumptions were defined that only apply in this form to a small number of patients presenting to a sleep medicine center. However, these general assumptions were necessary in order to achieve a stringent evaluation by ChatGPT and thus establish the comparability of the results to those of the board-certified sleep physician. Third, only those polysomnographic cases were designed that allowed little room for alternative therapy suggestions based on objective measurement data. In particular, subjective and anatomical factors must be taken into account for lower AHI ranges, as these are often decisive for a patient-specific therapy suggestion in such cases. Fourth, clinical symptoms, anatomical factors or sleep medicine questionnaires were not included in the design of the fictitious patients. This compromise was accepted in order to find out whether ChatGPT is fundamentally capable of making a correct diagnosis on the basis of specific, selected polysomnographic data and making a suitable therapy suggestion. Moreover, it should be noted that only cases of obstructive sleep apnea were evaluated in this study and other forms of sleep-related breathing disorders were not taken into account. In conclusion, the main limitation of this study is that only fictitious and basically very simplified sleep medicine cases were generated and subsequently used to demonstrate the potential of a ChatGPT-based evaluation. The transfer to clinical practice is therefore limited due to the often much greater complexity and individuality of sleep medicine cases.

Accepting these limitations, this is the first study to demonstrate the potential use of ChatGPT in the evaluation of polysomnographic data. Rather than the often discussed fear of replacing medical professionals, artificial intelligence-based clinical decision support and LLMs are tools to augment doctoral intelligence. The integration of augmented intelligence in medicine has tremendous potential to enhance human cognition without replacing human labor. However, it is important to emphasize the need to address concerns about transparency, accountability, and data reliability to ensure successful implementation in clinical practice [34]. In the field of sleep medicine, ChatGPT has the potential to perform preliminary evaluation of polysomnographic results, e.g. when qualified medical personnel are scarce. Although ChatGPT has high potential to support sleep physicians, it may face difficulties in correctly weighting information from anamnestic discussions or physical examinations to form a diagnosis and make a suitable therapy suggestion. Further studies need to focus on these considerations and should include subjective factors, such as clinical symptoms, sleep medicine questionnaires or the results of the physical examination. In addition, further studies should address critical aspects of the use of LLMs, e.g. ChatGPT, such as the mandatory protection of the personal data used. In this context, it has already been pointed out by others that regulatory oversight of LLMs is essential [4, 35].

## Conclusion

ChatGPT-4o and sleep physicians show a high level of agreement in terms of diagnosis and therapy suggestions based on fictitious polysomnographic results from simple patient cases. ChatGPT-4o shows interpretation shortcomings in cases of more complex polysomnographic result constellations. In conclusion, precise prompting of LLMs holds great potential to economize polysomnographic result interpretation in the future.

## Supplementary Information

Below is the link to the electronic supplementary material.

Supplementary file1 (PDF 732 kb)

## Data Availability

All data used is accessible in the supplementary material.
