# Supplementary material for: Chasing sleep physicians: ChatGPT-4o on the interpretation of polysomnographic results
Source: Eur Arch Otorhinolaryngol. 2024 Oct 20;282(3):1631–9. doi: 10.1007/s00405-024-08985-3 (PMC11890353; doi:10.1007/s00405-024-08985-3)
Supplement: Supplementary file 1 — Supplementary file1 (PDF 732 kb) [file 405_2024_8985_MOESM1_ESM.pdf]

Prompts, patients profiles and responses (translated bei DeepL (deep.com))

<u>Basic assumptions for patients 1-30:</u>

- No comorbidities
- No long-term medication
- First-time polysomnography to clarify a sleep-related breathing disorder due to clinical signs of (obstructive) sleep apnoea
- Polysomnography was performed technically correctly
- Supine position for at least 60 min of the TST

PROMPT:
The following basic assumptions apply to the following patient:
- There are no comorbidities.
- There is no long-term medication.
- There is a first polysomnography to clarify a sleep-related breathing disorder due to clinical signs of (obstructive) sleep apnoea.
- The polysomnography was performed technically correctly.
- The supine position is at least 60 min of the total time of sleep.

Order:
For the polysomnography data below, a diagnosis of obstructive sleep apnoea syndrome should be made. Sleep disorders such as central apnoea should not be taken into account, only the diagnosis of obstructive sleep apnoea syndrome is required. The answer should not exceed 200 characters.

The findings should be structured as follows:
Diagnosis: XX (If available, the sleep apnoea syndrome should be classified as "none", "mild", "moderate" and "severe").
Treatment recommendation: XX (aPAP therapy recommended / no aPAP therapy necessary).

**Personal details**	
Name	Patient 1
Sex	M
Age	65
Boday mass index	27
**Respiratory assessment**	
Apnea hypopnea index	3,8
Apnea index	0,4
Hypopnea index	3,4
Cumulative apnea and hypopnea duration	8:03
**Evaluation of puls oximetry**	
Oxygen desaturation index	5,4
Average oxygen saturation	95
T90	0,0
**Neurological evaluation**	
Total sleep time	354,0
Sleep efficacy	79,5
**Evaluation of body position**	
Ratio apnea hypopnea index in supine position to not supine position	2,70 : 1
**Evaluation of board-certified sleep physician**	
Diagnosis	No evidence of clinically relevant sleep apnoea.
Therapy	None

ChatGPT-4o:
Diagnosis: No obstructive sleep apnoea (AHI < 5).
Treatment recommendation: No aPAP therapy necessary.

**Personal details**	
Name	Patient 2
Sex	W
Age	49
Boday mass index	35
**Respiratory assessment**	
Apnea hypopnea index	7,4
Apnea index	1,2
Hypopnea index	6,2
Cumulative apnea and hypopnea duration	13:04
**Evaluation of puls oximetry**	
Oxygen desaturation index	7,0
Average oxygen saturation	95
T90	0,0
**Neurological evaluation**	
Total sleep time	317,3
Sleep efficacy	79,6
**Evaluation of body position**	
Ratio apnea hypopnea index in supine position to not supine position	4,86 : 1
**Evaluation of board-certified sleep physician**	
Diagnosis	Low-grade (supine-associated) obstructive sleep apnoea.
Therapy	None

<u>ChatGPT-4o:</u>
Diagnosis: Low-grade obstructive sleep apnoea syndrome
Treatment recommendation: No aPAP therapy necessary.

**Personal details**	
Name	Patient 3
Sex	M
Age	53
Boday mass index	40
**Respiratory assessment**	
Apnea hypopnea index	9,8
Apnea index	1,9
Hypopnea index	7,9
Cumulative apnea and hypopnea duration	24:52
**Evaluation of puls oximetry**	
Oxygen desaturation index	13,6
Average oxygen saturation	95
T90	0,4
**Neurological evaluation**	
Total sleep time	389,9
Sleep efficacy	85,5
**Evaluation of body position**	
Ratio apnea hypopnea index in supine position to not supine position	1,16 : 1
**Evaluation of board-certified sleep physician**	
Diagnosis	Minor obstructive sleep apnoea.
Therapy	None

<u>ChatGPT-4o:</u>
Diagnosis: Low-grade obstructive sleep apnoea syndrome.
Treatment recommendation: No aPAP therapy necessary.

Personal details	
Name	Patient 4
Sex	W
Age	34
Boday mass index	26
**Respiratory assessment**	
Apnea hypopnea index	5,2
Apnea index	0,4
Hypopnea index	4,8
Cumulative apnea and hypopnea duration	10:45
**Evaluation of puls oximetry**	
Oxygen desaturation index	2,2
Average oxygen saturation	94
T90	0,0
**Neurological evaluation**	
Total sleep time	345,9
Sleep efficacy	65,5
**Evaluation of body position**	
Ratio apnea hypopnea index in supine position to not supine position	5,10 : 0
**Evaluation of board-certified sleep physician**	
Diagnosis	Low-grade (supine-associated) obstructive sleep apnoea.
Therapy	None

ChatGPT-4o:
Diagnosis: Low-grade obstructive sleep apnoea syndrome (AHI 5.2/h)
Treatment recommendation: No aPAP therapy necessary.

**Personal details**	
Name	Patient 5
Sex	M
Age	72
Boday mass index	29
**Respiratory assessment**	
Apnea hypopnea index	8,8
Apnea index	0,5
Hypopnea index	8,3
Cumulative apnea and hypopnea duration	22:25
**Evaluation of puls oximetry**	
Oxygen desaturation index	8,7
Average oxygen saturation	94
T90	0,0
**Neurological evaluation**	
Total sleep time	441,9
Sleep efficacy	89,9
**Evaluation of body position**	
Ratio apnea hypopnea index in supine position to not supine position	7,70 : 1
**Evaluation of board-certified sleep physician**	
Diagnosis	Low-grade (supine-associated) obstructive sleep apnoea.
Therapy	None

<u>ChatGPT-4o:</u>
Diagnosis: Low-grade obstructive sleep apnoea syndrome
Treatment recommendation: No aPAP therapy necessary.

**Personal details**	
Name	Patient 6
Sex	W
Age	79
Boday mass index	24
**Respiratory assessment**	
Apnea hypopnea index	2,1
Apnea index	0,3
Hypopnea index	1,8
Cumulative apnea and hypopnea duration	7:38
**Evaluation of puls oximetry**	
Oxygen desaturation index	3,2
Average oxygen saturation	95
T90	0,0
**Neurological evaluation**	
Total sleep time	399,5
Sleep efficacy	81,3
**Evaluation of body position**	
Ratio apnea hypopnea index in supine position to not supine position	2,60 : 1
**Evaluation of board-certified sleep physician**	
Diagnosis	No evidence of clinically relevant sleep apnoea.
Therapy	None

<u>ChatGPT-4o:</u>
Diagnosis: No obstructive sleep apnoea syndrome (AHI < 5).
Treatment recommendation: No aPAP therapy necessary.

**Personal details**	
Name	Patient 7
Sex	M
Age	51
Boday mass index	25
**Respiratory assessment**	
Apnea hypopnea index	7,1
Apnea index	2,0
Hypopnea index	5,1
Cumulative apnea and hypopnea duration	13:12
**Evaluation of puls oximetry**	
Oxygen desaturation index	7,6
Average oxygen saturation	94
T90	0,3
**Neurological evaluation**	
Total sleep time	300,9
Sleep efficacy	90,7
**Evaluation of body position**	
Ratio apnea hypopnea index in supine position to not supine position	1,60 : 1
**Evaluation of board-certified sleep physician**	
Diagnosis	Minor obstructive sleep apnoea.
Therapy	None

<u>ChatGPT-4o:</u>
Diagnosis: Low-grade obstructive sleep apnoea syndrome
Treatment recommendation: No aPAP therapy necessary.

Personal details	
Name	Patient 8
Sex	W
Age	37
Boday mass index	28
**Respiratory assessment**	
Apnea hypopnea index	6,1
Apnea index	1,1
Hypopnea index	5,0
Cumulative apnea and hypopnea duration	12:51
**Evaluation of puls oximetry**	
Oxygen desaturation index	8,6
Average oxygen saturation	95
T90	0,2
**Neurological evaluation**	
Total sleep time	355,7
Sleep efficacy	93,5
**Evaluation of body position**	
Ratio apnea hypopnea index in supine position to not supine position	1,28 : 1
**Evaluation of board-certified sleep physician**	
Diagnosis	Minor obstructive sleep apnoea.
Therapy	None

ChatGPT-4o
Diagnosis: Low-grade obstructive sleep apnoea syndrome (AHI 6.1)
Treatment recommendation: No aPAP therapy necessary.

**Personal details**	
Name	Patient 9
Sex	M
Age	58
Boday mass index	23
**Respiratory assessment**	
Apnea hypopnea index	7,6
Apnea index	1,0
Hypopnea index	6,6
Cumulative apnea and hypopnea duration	12:58
**Evaluation of puls oximetry**	
Oxygen desaturation index	6,9
Average oxygen saturation	94
T90	0,0
**Neurological evaluation**	
Total sleep time	386,8
Sleep efficacy	98,8
**Evaluation of body position**	
Ratio apnea hypopnea index in supine position to not supine position	4,39 : 1
**Evaluation of board-certified sleep physician**	
Diagnosis	Low-grade (supine-associated) obstructive sleep apnoea.
Therapy	None

ChatGPT-4o:
Diagnosis: Low-grade obstructive sleep apnoea syndrome
Treatment recommendation: No aPAP therapy necessary.

**Personal details**	
Name	Patient 10
Sex	W
Age	61
Boday mass index	22
**Respiratory assessment**	
Apnea hypopnea index	3,6
Apnea index	0,6
Hypopnea index	3,0
Cumulative apnea and hypopnea duration	8:00
**Evaluation of puls oximetry**	
Oxygen desaturation index	4,1
Average oxygen saturation	95
T90	0,0
**Neurological evaluation**	
Total sleep time	407,2
Sleep efficacy	89,2
**Evaluation of body position**	
Ratio apnea hypopnea index in supine position to not supine position	2,00 : 1
**Evaluation of board-certified sleep physician**	
Diagnosis	No evidence of clinically relevant sleep apnoea.
Therapy	None

ChatGPT-4o:
Diagnosis: No obstructive sleep apnoea syndrome (AHI < 5).
Treatment recommendation: No aPAP therapy necessary.

**Personal details**	
Name	Patient 11
Sex	M
Age	28
Boday mass index	27
**Respiratory assessment**	
Apnea hypopnea index	17,5
Apnea index	7,9
Hypopnea index	9,5
Cumulative apnea and hypopnea duration	25:41
**Evaluation of puls oximetry**	
Oxygen desaturation index	13,5
Average oxygen saturation	94
T90	0,0
**Neurological evaluation**	
Total sleep time	388,4
Sleep efficacy	95,9
**Evaluation of body position**	
Ratio apnea hypopnea index in supine position to not supine position	16,00 : 1
**Evaluation of board-certified sleep physician**	
Diagnosis	Moderate (supine-accentuated) obstructive sleep apnoea.
Therapy	Initiation of aPAP therapy (5-9 mbar) and polysomnographic monitoring of the PAP setting 3 months after the start of PAP therapy.

<u>ChatGPT-4o:</u>
Diagnosis: Moderate obstructive sleep apnoea syndrome.
Treatment recommendation: aPAP therapy recommended.

**Personal details**	
Name	Patient 12
Sex	W
Age	37
Boday mass index	28
**Respiratory assessment**	
Apnea hypopnea index	19,8
Apnea index	1,8
Hypopnea index	18,0
Cumulative apnea and hypopnea duration	27:20
**Evaluation of puls oximetry**	
Oxygen desaturation index	34,2
Average oxygen saturation	94
T90	0,1
**Neurological evaluation**	
Total sleep time	333,9
Sleep efficacy	69,6
**Evaluation of body position**	
Ratio apnea hypopnea index in supine position to not supine position	2,99 : 1
**Evaluation of board-certified sleep physician**	
Diagnosis	Moderate (supine-accentuated) obstructive sleep apnoea.
Therapy	Initiation of aPAP therapy (5-9 mbar) and polysomnographic monitoring of the PAP setting 3 months after the start of PAP therapy.

ChatGPT-4o:
Diagnosis: Moderate obstructive sleep apnoea syndrome.
Treatment recommendation: aPAP therapy recommended.

**Personal details**	
Name	Patient 13
Sex	M
Age	43
Boday mass index	32
**Respiratory assessment**	
Apnea hypopnea index	22,6
Apnea index	2,4
Hypopnea index	20,2
Cumulative apnea and hypopnea duration	29:15
**Evaluation of puls oximetry**	
Oxygen desaturation index	26,3
Average oxygen saturation	94
T90	1,2
**Neurological evaluation**	
Total sleep time	374,9
Sleep efficacy	89,6
**Evaluation of body position**	
Ratio apnea hypopnea index in supine position to not supine position	1,26 : 1
**Evaluation of board-certified sleep physician**	
Diagnosis	Moderate obstructive sleep apnoea.
Therapy	Initiation of aPAP therapy (5-10 mbar) and polysomnographic monitoring of the PAP setting 3 months after the start of PAP therapy.

ChatGPT-4o:
Diagnosis: Moderate obstructive sleep apnoea syndrome (AHI 22.6/h)
Treatment recommendation: aPAP therapy recommended.

**Personal details**	
Name	Patient 14
Sex	W
Age	57
Boday mass index	30
**Respiratory assessment**	
Apnea hypopnea index	23,4
Apnea index	7,8
Hypopnea index	15,6
Cumulative apnea and hypopnea duration	41:12
**Evaluation of puls oximetry**	
Oxygen desaturation index	25,6
Average oxygen saturation	94
T90	0,5
**Neurological evaluation**	
Total sleep time	487,7
Sleep efficacy	95,8
**Evaluation of body position**	
Ratio apnea hypopnea index in supine position to not supine position	5,11 : 1
**Evaluation of board-certified sleep physician**	
Diagnosis	Moderate (supine-accentuated) obstructive sleep apnoea.
Therapy	Initiation of aPAP therapy (5-9 mbar) and polysomnographic monitoring of the PAP setting 3 months after the start of PAP therapy.

<u>ChatGPT-4o:</u>
Diagnosis: Moderate obstructive sleep apnoea syndrome
Treatment recommendation: aPAP therapy recommended.

**Personal details**	
Name	Patient 15
Sex	M
Age	49
Boday mass index	29
**Respiratory assessment**	
Apnea hypopnea index	25,3
Apnea index	2,0
Hypopnea index	23,1
Cumulative apnea and hypopnea duration	49:10
**Evaluation of puls oximetry**	
Oxygen desaturation index	22,4
Average oxygen saturation	94
T90	0,6
**Neurological evaluation**	
Total sleep time	385,6
Sleep efficacy	83,6
**Evaluation of body position**	
Ratio apnea hypopnea index in supine position to not supine position	1,08 : 1
**Evaluation of board-certified sleep physician**	
Diagnosis	Moderate obstructive sleep apnoea.
Therapy	Initiation of aPAP therapy (5-9 mbar) and polysomnographic monitoring of the PAP setting 3 months after the start of PAP therapy.

<u>ChatGPT-4o:</u>
Diagnosis: Moderate obstructive sleep apnoea syndrome
Treatment recommendation: aPAP therapy recommended.

Personal details	
Name	Patient 16
Sex	W
Age	32
Boday mass index	25
**Respiratory assessment**	
Apnea hypopnea index	26,3
Apnea index	8,6
Hypopnea index	17,7
Cumulative apnea and hypopnea duration	51:23
**Evaluation of puls oximetry**	
Oxygen desaturation index	23,4
Average oxygen saturation	94
T90	0,2
**Neurological evaluation**	
Total sleep time	436,9
Sleep efficacy	79,8
**Evaluation of body position**	
Ratio apnea hypopnea index in supine position to not supine position	1,89 : 1
**Evaluation of board-certified sleep physician**	
Diagnosis	Moderate obstructive sleep apnoea.
Therapy	Initiation of aPAP therapy (5-9 mbar) and polysomnographic monitoring of the PAP setting 3 months after the start of PAP therapy.

ChatGPT-4o:
Diagnosis: Moderate obstructive sleep apnoea syndrome (AHI 26.3/h).
Treatment recommendation: aPAP therapy recommended.

Personal details	
Name	Patient 17
Sex	M
Age	35
Boday mass index	23
**Respiratory assessment**	
Apnea hypopnea index	24,3
Apnea index	3,3
Hypopnea index	21,0
Cumulative apnea and hypopnea duration	44:51
**Evaluation of puls oximetry**	
Oxygen desaturation index	22,2
Average oxygen saturation	94
T90	8,8
**Neurological evaluation**	
Total sleep time	403,6
Sleep efficacy	90,6
**Evaluation of body position**	
Ratio apnea hypopnea index in supine position to not supine position	2,67 : 1
**Evaluation of board-certified sleep physician**	
Diagnosis	Moderate (supine-accentuated) obstructive sleep apnoea.
Therapy	Initiation of aPAP therapy (5-9 mbar) and polysomnographic monitoring of the PAP setting 3 months after the start of PAP therapy.

ChatGPT-4o:
Diagnosis: Moderate obstructive sleep apnoea syndrome (AHI 24.3/h)
Treatment recommendation: aPAP therapy recommended.

**Personal details**	
Name	Patient 18
Sex	W
Age	78
Boday mass index	31
**Respiratory assessment**	
Apnea hypopnea index	28,0
Apnea index	15,0
Hypopnea index	13,0
Cumulative apnea and hypopnea duration	49:55
**Evaluation of puls oximetry**	
Oxygen desaturation index	33,9
Average oxygen saturation	94
T90	23,3
**Neurological evaluation**	
Total sleep time	359,7
Sleep efficacy	66,9
**Evaluation of body position**	
Ratio apnea hypopnea index in supine position to not supine position	1,90 : 1
**Evaluation of board-certified sleep physician**	
Diagnosis	Moderate obstructive sleep apnoea.
Therapy	Initiation of aPAP therapy (5-9 mbar) and polysomnographic monitoring of the PAP setting 3 months after the start of PAP therapy.

ChatGPT-4o:
Diagnosis: Moderate obstructive sleep apnoea syndrome (AHI 28.0/h).
Therapy recommendation: aPAP therapy recommended.

Personal details	
Name	Patient 19
Sex	M
Age	64
Boday mass index	28
**Respiratory assessment**	
Apnea hypopnea index	28,2
Apnea index	5,1
Hypopnea index	23,1
Cumulative apnea and hypopnea duration	50:17
**Evaluation of puls oximetry**	
Oxygen desaturation index	24,2
Average oxygen saturation	94
T90	3,6
**Neurological evaluation**	
Total sleep time	323,5
Sleep efficacy	86,0
**Evaluation of body position**	
Ratio apnea hypopnea index in supine position to not supine position	1,40 : 1
**Evaluation of board-certified sleep physician**	
Diagnosis	Moderate obstructive sleep apnoea.
Therapy	Initiation of aPAP therapy (5-9 mbar) and polysomnographic monitoring of the PAP setting 3 months after the start of PAP therapy.

ChatGPT-4o:
Diagnosis: Moderate obstructive sleep apnoea syndrome
Treatment recommendation: aPAP therapy recommended.

**Personal details**	
Name	Patient 20
Sex	W
Age	59
Boday mass index	27
**Respiratory assessment**	
Apnea hypopnea index	29,5
Apnea index	4,0
Hypopnea index	25,5
Cumulative apnea and hypopnea duration	53:34
**Evaluation of puls oximetry**	
Oxygen desaturation index	20,5
Average oxygen saturation	94
T90	0,1
**Neurological evaluation**	
Total sleep time	345,6
Sleep efficacy	82,6
**Evaluation of body position**	
Ratio apnea hypopnea index in supine position to not supine position	1,00 : 1
**Evaluation of board-certified sleep physician**	
Diagnosis	Moderate obstructive sleep apnoea.
Therapy	Initiation of aPAP therapy (5-9 mbar) and polysomnographic monitoring of the PAP setting 3 months after the start of PAP therapy.

ChatGPT-4o:
Diagnosis: Moderate obstructive sleep apnoea syndrome
Treatment recommendation: aPAP therapy recommended.

Personal details	
Name	Patient 21
Sex	M
Age	40
Boday mass index	30
**Respiratory assessment**	
Apnea hypopnea index	82,5
Apnea index	80,4
Hypopnea index	2,1
Cumulative apnea and hypopnea duration	4:12:41
**Evaluation of puls oximetry**	
Oxygen desaturation index	78,2
Average oxygen saturation	91
T90	31,8
**Neurological evaluation**	
Total sleep time	365,5
Sleep efficacy	95,8
**Evaluation of body position**	
Ratio apnea hypopnea index in supine position to not supine position	1,25 : 1
**Evaluation of board-certified sleep physician**	
Diagnosis	Severe obstructive sleep apnoea.
Therapy	Initiation of aPAP therapy (5-9 mbar) and polysomnographic monitoring of the PAP setting 3 months after the start of PAP therapy.

ChatGPT-4o
Diagnosis: Severe obstructive sleep apnoea syndrome
Treatment recommendation: aPAP therapy recommended.

**Personal details**	
Name	Patient 22
Sex	W
Age	47
Boday mass index	29
**Respiratory assessment**	
Apnea hypopnea index	32,0
Apnea index	5,4
Hypopnea index	26,6
Cumulative apnea and hypopnea duration	51:28
**Evaluation of puls oximetry**	
Oxygen desaturation index	29,8
Average oxygen saturation	94
T90	4,1
**Neurological evaluation**	
Total sleep time	366,7
Sleep efficacy	68,9
**Evaluation of body position**	
Ratio apnea hypopnea index in supine position to not supine position	1,95 : 1
**Evaluation of board-certified sleep physician**	
Diagnosis	Severe obstructive sleep apnoea.
Therapy	Initiation of aPAP therapy (5-9 mbar) and polysomnographic monitoring of the PAP setting 3 months after the start of PAP therapy.

ChatGPT-4o:
Diagnosis: Moderate obstructive sleep apnoea syndrome (AHI 32.0/h)
Treatment recommendation: aPAP therapy recommended.

Personal details	
Name	Patient 23
Sex	M
Age	63
Boday mass index	32
**Respiratory assessment**	
Apnea hypopnea index	30,8
Apnea index	7,4
Hypopnea index	23,4
Cumulative apnea and hypopnea duration	59:49
**Evaluation of puls oximetry**	
Oxygen desaturation index	26,8
Average oxygen saturation	94
T90	1,8
**Neurological evaluation**	
Total sleep time	375,9
Sleep efficacy	58,9
**Evaluation of body position**	
Ratio apnea hypopnea index in supine position to not supine position	3,52 : 1
**Evaluation of board-certified sleep physician**	
Diagnosis	Severe (supine position-accentuated) obstructive sleep apnoea.
Therapy	Initiation of aPAP therapy (5-10 mbar) and polysomnographic monitoring of the PAP setting 3 months after the start of PAP therapy.

ChatGPT-4o
Diagnosis: Severe obstructive sleep apnoea syndrome
Treatment recommendation: aPAP therapy recommended.

Personal details	
Name	Patient 24
Sex	W
Age	32
Boday mass index	29
**Respiratory assessment**	
Apnea hypopnea index	33,3
Apnea index	3,3
Hypopnea index	30,0
Cumulative apnea and hypopnea duration	1:00:54
**Evaluation of puls oximetry**	
Oxygen desaturation index	31,1
Average oxygen saturation	94
T90	2,3
**Neurological evaluation**	
Total sleep time	432,5
Sleep efficacy	84,8
**Evaluation of body position**	
Ratio apnea hypopnea index in supine position to not supine position	1,05 : 1
**Evaluation of board-certified sleep physician**	
Diagnosis	Severe obstructive sleep apnoea.
Therapy	Initiation of aPAP therapy (5-9 mbar) and polysomnographic monitoring of the PAP setting 3 months after the start of PAP therapy.

ChatGPT-4o:
Diagnosis: Severe obstructive sleep apnoea syndrome (AHI 33.3/h).
Treatment recommendation: aPAP therapy recommended.

Personal details	
Name	Patient 25
Sex	M
Age	62
Boday mass index	26
**Respiratory assessment**	
Apnea hypopnea index	31,9
Apnea index	8,8
Hypopnea index	23,1
Cumulative apnea and hypopnea duration	1:07:33
**Evaluation of puls oximetry**	
Oxygen desaturation index	29,8
Average oxygen saturation	94
T90	2,0
**Neurological evaluation**	
Total sleep time	344,9
Sleep efficacy	79,9
**Evaluation of body position**	
Ratio apnea hypopnea index in supine position to not supine position	2,08 : 1
**Evaluation of board-certified sleep physician**	
Diagnosis	Severe (supine position-accentuated) obstructive sleep apnoea.
Therapy	Initiation of aPAP therapy (5-9 mbar) and polysomnographic monitoring of the PAP setting 3 months after the start of PAP therapy.

ChatGPT-4o:
Diagnosis: Severe obstructive sleep apnoea syndrome (AHI: 31.9/h)
Treatment recommendation: aPAP therapy recommended.

**Personal details**	
Name	Patient 26
Sex	W
Age	19
Boday mass index	33
**Respiratory assessment**	
Apnea hypopnea index	59,2
Apnea index	21,2
Hypopnea index	38,0
Cumulative apnea and hypopnea duration	2:13:42
**Evaluation of puls oximetry**	
Oxygen desaturation index	60,1
Average oxygen saturation	91
T90	3,1
**Neurological evaluation**	
Total sleep time	391,6
Sleep efficacy	95,3
**Evaluation of body position**	
Ratio apnea hypopnea index in supine position to not supine position	3,75 : 1
**Evaluation of board-certified sleep physician**	
Diagnosis	Severe (supine position-accentuated) obstructive sleep apnoea.
Therapy	Initiation of aPAP therapy (5-10 mbar) and polysomnographic monitoring of the PAP setting 3 months after the start of PAP therapy.

<u>ChatGPT-4o:</u>
Diagnosis: Severe obstructive sleep apnoea syndrome
Treatment recommendation: aPAP therapy recommended.

**Personal details**	
Name	Patient 27
Sex	M
Age	50
Boday mass index	31
**Respiratory assessment**	
Apnea hypopnea index	48,5
Apnea index	28,0
Hypopnea index	20,5
Cumulative apnea and hypopnea duration	1:42:10
**Evaluation of puls oximetry**	
Oxygen desaturation index	53,8
Average oxygen saturation	92
T90	0,6
**Neurological evaluation**	
Total sleep time	441,8
Sleep efficacy	88,0
**Evaluation of body position**	
Ratio apnea hypopnea index in supine position to not supine position	1,60 : 1
**Evaluation of board-certified sleep physician**	
Diagnosis	Severe obstructive sleep apnoea.
Therapy	Initiation of aPAP therapy (5-9 mbar) and polysomnographic monitoring of the PAP setting 3 months after the start of PAP therapy.

ChatGPT-4o:
Diagnosis: Severe obstructive sleep apnoea syndrome
Treatment recommendation: aPAP therapy recommended.

**Personal details**	
Name	Patient 28
Sex	W
Age	26
Boday mass index	22
**Respiratory assessment**	
Apnea hypopnea index	30,9
Apnea index	16,8
Hypopnea index	14,1
Cumulative apnea and hypopnea duration	52:02
**Evaluation of puls oximetry**	
Oxygen desaturation index	27,7
Average oxygen saturation	94
T90	0,0
**Neurological evaluation**	
Total sleep time	375,8
Sleep efficacy	77,6
**Evaluation of body position**	
Ratio apnea hypopnea index in supine position to not supine position	1,60 : 1
**Evaluation of board-certified sleep physician**	
Diagnosis	Severe obstructive sleep apnoea.
Therapy	Initiation of aPAP therapy (5-9 mbar) and polysomnographic monitoring of the PAP setting 3 months after the start of PAP therapy.

<u>ChatGPT-4o:</u>
Diagnosis: Severe obstructive sleep apnoea syndrome
Treatment recommendation: aPAP therapy recommended.

Personal details	
Name	Patient 29
Sex	M
Age	70
Boday mass index	30
**Respiratory assessment**	
Apnea hypopnea index	35,5
Apnea index	12,2
Hypopnea index	23,3
Cumulative apnea and hypopnea duration	1:09:13
**Evaluation of puls oximetry**	
Oxygen desaturation index	34,2
Average oxygen saturation	94
T90	4,3
**Neurological evaluation**	
Total sleep time	385,6
Sleep efficacy	81,3
**Evaluation of body position**	
Ratio apnea hypopnea index in supine position to not supine position	6,00 : 1
**Evaluation of board-certified sleep physician**	
Diagnosis	Severe (supine position-accentuated) obstructive sleep apnoea.
Therapy	Initiation of aPAP therapy (5-9 mbar) and polysomnographic monitoring of the PAP setting 3 months after the start of PAP therapy.

ChatGPT-4o:
Diagnosis: Severe obstructive sleep apnoea syndrome
Treatment recommendation: aPAP therapy recommended.

**Personal details**	
Name	Patient 30
Sex	W
Age	49
Boday mass index	32
**Respiratory assessment**	
Apnea hypopnea index	62,5
Apnea index	60,4
Hypopnea index	2,1
Cumulative apnea and hypopnea duration	2:39:27
**Evaluation of puls oximetry**	
Oxygen desaturation index	61,2
Average oxygen saturation	91
T90	29,8
**Neurological evaluation**	
Total sleep time	400,5
Sleep efficacy	80,8
**Evaluation of body position**	
Ratio apnea hypopnea index in supine position to not supine position	1,80 : 1
**Evaluation of board-certified sleep physician**	
Diagnosis	Severe obstructive sleep apnoea.
Therapy	Initiation of aPAP therapy (5-10 mbar) and polysomnographic monitoring of the PAP setting 3 months after the start of PAP therapy.

<u>ChatGPT-4o:</u>
Diagnosis: Severe obstructive sleep apnoea syndrome
Treatment recommendation: aPAP therapy recommended.

Basic assumptions for all patients 31-40:

- \- No comorbidities
- \- No long-term medication
- \- At least mild obstructive sleep apnoea already confirmed by external polysomnography/polygraphy
- \- External polysomnography/polygraphy was performed due to clinical signs of (obstructive) sleep apnoea
- \- PAP therapy has already been initiated and trialled
- \- PAP therapy is not tolerated and therefore rejected by patients
- \- Various mask fits have already been trialled (e.g. full face mask, nasal mask, nasal cushion mask)
- \- Presentation in our sleep laboratory for renewed polysomnography
- \- Polysomnography was performed technically correctly
- \- Supine position for at least 60 min of TST (total time of sleep)

PROMT

The following basic assumptions apply to the following patient:
- No comorbidities
- No long-term medication
- At least mild obstructive sleep apnoea already confirmed by external polysomnography/polygraphy
- External polysomnography/polygraphy was performed due to clinical signs of (obstructive) sleep apnoea
- PAP therapy has already been initiated and trialled
- PAP therapy is not tolerated and therefore rejected by patients
- Various mask fits have already been trialled (e.g. full face mask, nasal mask, nasal cushion mask)
- Presentation in our sleep laboratory for renewed polysomnography
- Polysomnography was performed technically correctly
- Supine position for at least 60 min of TST (total time of sleep)

Task:
For the polysomnography data below, a diagnosis of obstructive sleep apnoea syndrome is to be made. Sleep disorders such as central apnoea should not be taken into account, only the diagnosis of obstructive sleep apnoea syndrome is required. The answer should not exceed 200 characters.
The findings should be structured as follows:
Diagnosis: XX (If present, the sleep apnoea syndrome should be classified as "none", "mild", "moderate" and "severe").
Therapy recommendation: If PAP intolerance exists: XX (Alternative to PAP therapy, as this was not tolerated)

**Personal details**	
Name	Patient 31
Sex	M
Age	61
Boday mass index	26
**Respiratory assessment**	
Apnea hypopnea index	9,8
Apnea index	0,4
Hypopnea index	9,4
Cumulative apnea and hypopnea duration	25:03
**Evaluation of puls oximetry**	
Oxygen desaturation index	9,4
Average oxygen saturation	95
T90	0,0
**Neurological evaluation**	
Total sleep time	349,0
Sleep efficacy	79,5
**Evaluation of body position**	
Ratio apnea hypopnea index in supine position to not supine position	1,70 : 1
**Evaluation of board-certified sleep physician**	
Diagnosis	Minor obstructive sleep apnoea.
Therapy	In case of PAP intolerance: Discussion of the treatment Agenatives mandibular advancement splint or OSA surgery with suitable obstructive anatomy. Presentation in the sleep laboratory consultation.

ChatGPT-4o:
Diagnosis: Low-grade obstructive sleep apnoea syndrome
Treatment recommendation: In case of existing PAP intolerance: testing of Alternative treatment methods such as mandibular advancement splint or lateral position therapy.

**Personal details**	
Name	Patient 32
Sex	W
Age	48
Boday mass index	29
**Respiratory assessment**	
Apnea hypopnea index	12,4
Apnea index	1,2
Hypopnea index	1,2
Cumulative apnea and hypopnea duration	38:04
**Evaluation of puls oximetry**	
Oxygen desaturation index	12,0
Average oxygen saturation	95
T90	0,0
**Neurological evaluation**	
Total sleep time	319,2
Sleep efficacy	79,6
**Evaluation of body position**	
Ratio apnea hypopnea index in supine position to not supine position	4,86 : 1
**Evaluation of board-certified sleep physician**	
Diagnosis	Low-grade (supine-associated) obstructive sleep apnoea.
Therapy	In case of PAP intolerance: Attempt therapy by preventing supine position (e.g. supine position prevention waistcoat).

<u>ChatGPT-4o</u>
Diagnosis: Moderate obstructive sleep apnoea syndrome (AHI 12.4/h)
Treatment recommendation: If PAP intolerance is present: Position-based therapy (e.g. avoidance of supine position) and/or oral apnoea splints.

Personal details	
Name	Patient 33
Sex	M
Age	54
Boday mass index	25
**Respiratory assessment**	
Apnea hypopnea index	13,8
Apnea index	1,8
Hypopnea index	12,0
Cumulative apnea and hypopnea duration	54:52
**Evaluation of puls oximetry**	
Oxygen desaturation index	13,6
Average oxygen saturation	94
T90	0,3
**Neurological evaluation**	
Total sleep time	392,9
Sleep efficacy	85,5
**Evaluation of body position**	
Ratio apnea hypopnea index in supine position to not supine position	1,23 : 1
**Evaluation of board-certified sleep physician**	
Diagnosis	Minor obstructive sleep apnoea.
Therapy	In case of PAP intolerance: Discussion of the treatment Agenatives mandibular advancement splint or OSA surgery with suitable obstructive anatomy. Presentation in the sleep laboratory consultation.

ChatGPT-4o
Diagnosis: Moderate obstructive sleep apnoea syndrome (AHI 13.8/h).
Treatment recommendation: If PAP intolerance is present: try a mandibular advancement splint (mandibular advancement splint) or positional therapy.

Personal details	
Name	Patient 34
Sex	W
Age	38
Boday mass index	27
**Respiratory assessment**	
Apnea hypopnea index	9,2
Apnea index	0,4
Hypopnea index	8,8
Cumulative apnea and hypopnea duration	13:45
**Evaluation of puls oximetry**	
Oxygen desaturation index	6,2
Average oxygen saturation	94
T90	0,0
**Neurological evaluation**	
Total sleep time	365,9
Sleep efficacy	65,5
**Evaluation of body position**	
Ratio apnea hypopnea index in supine position to not supine position	5,10 : 0
**Evaluation of board-certified sleep physician**	
Diagnosis	Low-grade (supine-associated) obstructive sleep apnoea.
Therapy	In case of PAP intolerance: Attempt therapy by preventing supine position (e.g. supine position prevention waistcoat).

ChatGPT-4o
Diagnosis: Low-grade obstructive sleep apnoea syndrome
Treatment recommendation: If PAP intolerance is present: Try positional therapy or a mandibular advancement splint.

**Personal details**	
Name	Patient 35
Sex	M
Age	72
Boday mass index	28
**Respiratory assessment**	
Apnea hypopnea index	12,8
Apnea index	0,5
Hypopnea index	12,3
Cumulative apnea and hypopnea duration	38:25
**Evaluation of puls oximetry**	
Oxygen desaturation index	12,7
Average oxygen saturation	94
T90	0,0
**Neurological evaluation**	
Total sleep time	431,9
Sleep efficacy	88,9
**Evaluation of body position**	
Ratio apnea hypopnea index in supine position to not supine position	1,70 : 1
**Evaluation of board-certified sleep physician**	
Diagnosis	Minor obstructive sleep apnoea.
Therapy	In case of PAP intolerance: Discussion of the treatment Agenatives mandibular advancement splint or OSA surgery with suitable obstructive anatomy. Presentation in the sleep laboratory consultation.

<u>ChatGPT-4o</u>
Diagnosis: Moderate obstructive sleep apnoea syndrome
Treatment recommendation: If PAP intolerance is present: try a mandibular advancement splint (mandibular advancement splint) or position-dependent therapy (e.g. avoidance of supine position).

**Personal details**	
Name	Patient 36
Sex	W
Age	39
Boday mass index	31
**Respiratory assessment**	
Apnea hypopnea index	59,3
Apnea index	21,3
Hypopnea index	38,0
Cumulative apnea and hypopnea duration	2:07:32
**Evaluation of puls oximetry**	
Oxygen desaturation index	61,1
Average oxygen saturation	91
T90	3,2
**Neurological evaluation**	
Total sleep time	393,7
Sleep efficacy	95,3
**Evaluation of body position**	
Ratio apnea hypopnea index in supine position to not supine position	1,75 : 1
**Evaluation of board-certified sleep physician**	
Diagnosis	Severe obstructive sleep apnoea.
Therapy	In case of PAP intolerance: Evaluation of treatment using neurostimulation of the hypoglossal nerve. Supplementary video sleep endoscopy necessary.

<u>ChatGPT-4o</u>
Diagnosis: Severe obstructive sleep apnoea syndrome (AHI: 59.3/h)
Treatment recommendation: If PAP intolerance is present: consider mandibular advancement splint or surgical measures (e.g. uvulopalatopharyngoplasty).

**Personal details**	
Name	Patient 37
Sex	M
Age	51
Boday mass index	30
**Respiratory assessment**	
Apnea hypopnea index	47,5
Apnea index	27,0
Hypopnea index	20,5
Cumulative apnea and hypopnea duration	1:32:10
**Evaluation of puls oximetry**	
Oxygen desaturation index	52,8
Average oxygen saturation	92
T90	0,5
**Neurological evaluation**	
Total sleep time	431,8
Sleep efficacy	87,3
**Evaluation of body position**	
Ratio apnea hypopnea index in supine position to not supine position	1,60 : 1
**Evaluation of board-certified sleep physician**	
Diagnosis	Severe obstructive sleep apnoea.
Therapy	In case of PAP intolerance: Evaluation of treatment using neurostimulation of the hypoglossal nerve. Supplementary video sleep endoscopy necessary.

ChatGPT-4o
Diagnosis: Severe obstructive sleep apnoea syndrome (AHI 47.5/h)
Treatment recommendation: If PAP intolerance is present: consider mandibular advancement splint, position-dependent therapy or surgical options.

Personal details	
Name	Patient 38
Sex	W
Age	27
Boday mass index	26
**Respiratory assessment**	
Apnea hypopnea index	38,9
Apnea index	16,8
Hypopnea index	22,1
Cumulative apnea and hypopnea duration	1:02:02
**Evaluation of puls oximetry**	
Oxygen desaturation index	37,7
Average oxygen saturation	94
T90	0,2
**Neurological evaluation**	
Total sleep time	377,8
Sleep efficacy	79,6
**Evaluation of body position**	
Ratio apnea hypopnea index in supine position to not supine position	1,60 : 1
**Evaluation of board-certified sleep physician**	
Diagnosis	Severe obstructive sleep apnoea.
Therapy	In case of PAP intolerance: Evaluation of treatment using neurostimulation of the hypoglossal nerve. Supplementary video sleep endoscopy necessary.

ChatGPT-4o
Diagnosis: Severe obstructive sleep apnoea (AHI 38.9/h)
Treatment recommendation: If PAP intolerance is present: Mandibular protrusion splint, positional therapy, weight-reducing measures, ENT clarification for surgical treatment.

**Personal details**	
Name	Patient 39
Sex	W
Age	72
Boday mass index	32
**Respiratory assessment**	
Apnea hypopnea index	37,5
Apnea index	14,2
Hypopnea index	23,3
Cumulative apnea and hypopnea duration	1:23:13
**Evaluation of puls oximetry**	
Oxygen desaturation index	36,2
Average oxygen saturation	94
T90	4,4
**Neurological evaluation**	
Total sleep time	382,8
Sleep efficacy	81,5
**Evaluation of body position**	
Ratio apnea hypopnea index in supine position to not supine position	1,97 : 1
**Evaluation of board-certified sleep physician**	
Diagnosis	Severe obstructive sleep apnoea.
Therapy	In case of PAP intolerance: Evaluation of treatment using neurostimulation of the hypoglossal nerve. Supplementary video sleep endoscopy necessary.

<u>ChatGPT-4o</u>
Diagnosis: Severe obstructive sleep apnoea syndrome
Treatment recommendation: If PAP intolerance is present: Mandibular protrusion splint (MPS) or surgical measures such as uvulopalatopharyngoplasty (UPPP) should be considered.

Personal details	
Name	Patient 40
Sex	W
Age	46
Boday mass index	31
**Respiratory assessment**	
Apnea hypopnea index	63,5
Apnea index	61,4
Hypopnea index	2,1
Cumulative apnea and hypopnea duration	2:42:17
**Evaluation of puls oximetry**	
Oxygen desaturation index	62,2
Average oxygen saturation	91
T90	25,8
**Neurological evaluation**	
Total sleep time	402,5
Sleep efficacy	80,8
**Evaluation of body position**	
Ratio apnea hypopnea index in supine position to not supine position	1,80 : 1
**Evaluation of board-certified sleep physician**	
Diagnosis	Severe obstructive sleep apnoea.
Therapy	In case of PAP intolerance: Evaluation of treatment using neurostimulation of the hypoglossal nerve. Supplementary video sleep endoscopy necessary.

ChatGPT-4o:
Diagnosis: Severe obstructive sleep apnoea syndrome
Treatment recommendation: If PAP intolerance is present: Agenatives to PAP therapy may include a mandibular advancement splint or positioning therapy. Other options are surgical therapy or weight reduction.

Exemplary Prompt for Patient 1
(translated bei DeepL (deep.com), original prompts were in German language)

The following basic assumptions apply to the following patient:
- There are no comorbidities.
- There is no long-term medication.
- There is a first polysomnography to clarify a sleep-related breathing disorder due to clinical signs of (obstructive) sleep apnoea.
- The polysomnography was performed technically correctly.
- The supine position is at least 60 min of the total time of sleep.

Order:
For the polysomnography data below, a diagnosis of obstructive sleep apnoea syndrome should be made. Sleep disorders such as central apnoea should not be taken into account, only the diagnosis of obstructive sleep apnoea syndrome is required. The answer should not exceed 200 characters.

The findings should be structured as follows:
Diagnosis: XX (If available, the sleep apnoea syndrome should be classified as "none", "mild", "moderate" and "severe").
Treatment recommendation: XX (aPAP therapy recommended / no aPAP therapy necessary).

Personal details
Name Patient 1
Sex M
Age 65
Boday mass index 27
Respiratory assessment
Apnea hypopnea index 3,8
Apnea index 0,4
Hypopnea index 3,4
Cumulative apnea and hypopnea duration 8:03
Evaluation of puls oximetry
Oxygen desaturation index 5,4
Average oxygen saturation 95
T90 0,0
Neurological evaluation
Total sleep time 354,0
Sleep efficacy 79,5
Evaluation of body position
Ratio apnea hypopnea index in supine position to not supine position 2,70 : 1
